# Universal Method for Producing Reduced Graphene Oxide/Gold Nanoparticles Composites with Controlled Density of Grafting and Long-Term Stability

**DOI:** 10.3390/nano9040602

**Published:** 2019-04-11

**Authors:** Piotr Szustakiewicz, Natalia Kołsut, Aneta Leniart, Wiktor Lewandowski

**Affiliations:** 1 Faculty of Chemistry, University of Warsaw, 1 Pasteura st., 02-093 Warsaw, Poland; p.szustakiewicz@chem.uw.edu.pl (P.S.); n.kolsut@student.uw.edu.pl (N.K.); anetaleniart93@gmail.com (A.L.); 2 Faculty of Physics, University of Warsaw, 5 Pasteura st., 02-093 Warsaw, Poland; 3 CICbiomaGUNE, Paseo de Miramón 182, 20014 Donostia−San Sebastián, Spain

**Keywords:** graphene, reduced graphene oxide, nanoparticles, anisotropic, nanotriangles, nanostars, nanorods, bipyramids

## Abstract

In this study, we report a universal approach allowing the non-covalent deposition of gold nanoparticles on reduced graphene oxide surface in a controlled fashion. We used a modified Hummers method to obtain graphene oxide, which then underwent surficial functionalization with carboxyl moieties coupled with simultaneous reduction. Nanoparticles were synthesized ex-situ and capped with a thiolated poly-ethylene glycol (PEG) ligand. The interactions between the surface of modified graphene oxide and nanoparticle ligands enabled the formation of stable hybrid graphene-nanoparticles materials in the aqueous phase. Using this technique, we were able to cover the surface of graphene with gold nanoparticles of different shapes (spheres, rods, triangles, stars, and bipyramids), broad range of sizes (from 5 nm to 100 nm) and controlled grafting densities. Moreover, materials obtained with this strategy exhibited long-term stability, which coupled with the versatility and facility of preparation, makes our technique appealing in the light of increasing demand for new graphene-based hybrid nanostructures.

## 1. Introduction

In 2004, Novoselov et al. [1] isolated and characterized single-layered graphene from graphite, giving rise to a new field of research—2D materials. Since then, a wide range of graphene-based 2D materials have emerged with applications in sensing [2], energy storage [3], and conversion [4] or catalysis [5]. It is the graphene’s extraordinary electrical and optical properties, mechanical strength, exceptional thermal conductivity, large surface area, and chemical inertness [6] that allows for such wide range of applications. However, to fully exploit its applicative potential, facile and cheap protocols for incorporating graphene into composite materials are required [7]. 

Currently, a huge library of different graphene-based materials exists and is rapidly expanded [8,9]. These composites can be divided by the role of graphene. On the one hand we can use graphene as a dopant, most notable examples includes improving polymer-based materials properties by doping it with graphene [10,11]. On the other hand, graphene can be utilized as a scaffold for other structures. In this area, graphene/nanoparticle (G/NP) composites stand out due to emerging applications in highly desired areas of oxygen reduction and evolution catalysis [9,12], photocatalytic hydrogen production [13], supercapacitors [14], and lithium energy storage devices [13]. G/NP materials have also been utilized as biosensors, enabling detection of trace amount of analyte molecules through electrochemical sensing [15] or surface-enhanced Raman scattering (SERS) [16]. 

Promising properties, along with high application potential, have led to the development of various techniques of preparing G/NP composite materials, which can be divided into two main categories: (1) an in-situ approach, in which nanoparticles are grown directly on a graphene/graphene derivative surface, offers fast and efficient method of decorating graphene. The downside of these methods is limited control over the size, shape and distribution of the nanoparticles [4]; and (2) an alternative ex-situ approach, in which nanoparticles are synthesized separately and then deposited onto a graphene surface. This allows for precise control over the shape and size of nanoparticles attached to graphene, which translates to the ability of adjusting characteristic of the final product for specific applicatory requirements. For example, He et al. [17] prepared G/NP composites using both in-situ growth of metal nanoparticles and deposition of pre-synthesized nanoparticles onto graphene surface, showing ex-situ approach superiority in terms of control over size and distribution of nanoparticles. Unfortunately, often the process of composite fabrication requires optimization for each type of nanoparticle separately [18]. Thus, developing an ex-situ approach suitable for different types of nanoparticles would be beneficial.

Several successful ex-situ approaches have been proposed. They can be categorized according to the interactions governing composite formation, that is covalent and non-covalent nanoparticles’ attachment. Formation of amide bonds is a popular choice in the covalent approach—along this line, for example CdS quantum dots [19] or Fe_2_O_3_ paramagnetic nanoparticles [20] were grafted onto graphene. However, this approach requires introducing an additional step in the preparation protocol. Non-covalent strategy is usually less complex. For example, Hong et al. attached positively charged gold nanoparticles to 1-pyrene butyric acid-modified graphene through electrostatic interactions [21]. Recently, Dimiev et al. utilized hydrophobic forces to attach magnetite nanoparticles to graphene oxide (GO), resulting in composites with unique magneto-optical behavior [22]. An elegant approach was also shown by Liu et al., who first introduced bovine serum albumin (BSA) to GO surface, and then deposited various spherical nanoparticles with controlled grafting density [23]. Thus, towards achieving a simple, versatile, and precisely controlled method to stable G/NP composites, it seems reasonable to follow a non-covalent, ex-situ approach that does not require the presence of additional entities. 

To achieve these goals, while still maintaining maximum control over final product properties, we report a universal, precisely controlled, ex-situ method of G/NP composite fabrication. Firstly, graphene oxide, obtained via the modified Hummers method, was chemically decorated with amide moieties in the Claisen rearrangement reaction. This process was coupled with simultaneous reduction of oxygen moieties and led to amide-modified reduced graphene oxide (ARGO), which was further subjected to hydrolysis, giving the final product of carboxyl-modified-reduced graphene oxide (CRGO) [24]. Separately, nanoparticles with various shapes (spheres, triangles, rods, stars, and bipyramids) and sizes were synthesized using literature protocols and further modified with thiol-ended polyethylene glycol ligands to ensure colloidal stability and chemical compatibility with CRGO. Finally, we show that the performed chemical modifications ensure non-covalent interactions between nanomaterials, which enabled us to deposit various types of nanoparticles onto graphene’s surface in a controlled manner.

## 2. Materials and Methods

### 2.1. Materials

Tetrachloroauric acid (HAuCl_4_ ≥ 99%), hexadecyltrimethylammonium chloride (CTAC, 25 wt% in water), hexadecyltrimethylammonium bromide (CTAB, ≥99%), citric acid (≥99.5%), sodium borohydride (NaBH_4_), silver nitrate (AgNO_3_, ≥99%), hydrochloric acid (HCl, 37%), L-ascorbic acid (AA, ≥99%), sodium iodide (99.999% trace metals basis), trisodium citrate dehydrate, dodecylamine (≥99%), cyclohexane (99.5%), formaldehyde (37 wt% in H_2_O, 10–15% methanol as stabilizer), poly(ethylene glycol) methyl ether thiol (MW = 6 k; MW = 2 k), bis(2-methoxyethyl) ether (99.5%), N,N-dimethylacetamide dimethyl acetal (90%), graphite < 20 µm, sulfuric acid (95–98%), potassium permanganate (≥99%), hydrogen peroxide solution (30 wt% in water), potassium hydroxide pellets (≥85%), potassium bromide (Infrared spectroscopy - IR grade, ≥99%), chloroform (≥99.5%), acetone (≥99.9%), ethanol (95%) were purchased from Sigma-Aldrich. All chemicals were used without further purification. Milli-Q water was used in all experiments.

### 2.2. Gold Nanospheres (AuNP) Synthesis

Synthesis of gold nanospheres was carried out according to the Wang method [25]. Briefly, 750 mg of dodecylamine was dispersed in 25 mL cyclohexane and 6 mL of 37% formaldehyde solution was added. The mixture was stirred for 10 min at 25 °C, followed with separation of an organic phase. The organic phase was washed two times with water. Next, 10 mL of an aqueous solution of HAuCl_4_ (1 g HAuCl_4_ in 250 mL H_2_O) was added to the organic phase under vigorous stirring and the mixture was stirred for 40 min, after which organic phase containing gold nanoparticles was separated. Before further modifications, nanoparticles were precipitated by adding ethanol until suspension turbidity was observed, then centrifuged (7000 RPM over 5 min), and re-dispersed in cyclohexane.

### 2.3. Gold Nanorods (AuNR) Preparation

Synthesis of gold nanorods was carried out according to the seed-mediated growth method published by Guyot-Sionnest [26]. For seed preparation, 25 µL of a 0.05 M HAuCl_4_ solution was added to 4.7 mL of 0.1 M CTAB solution. After 5 min, 300 µL of a freshly prepared 0.01 M NaBH_4_ solution was injected under vigorous stirring. Subsequently, 120 µL of seeds solution was added to 10 mL of the growth solution containing CTAB (0.1 M), HAuCl_4_ (0.5 mM), AgNO_3_ (0.04 mM) and ascorbic acid (0.75 mM). The mixture was left undisturbed at 30 °C for 2 h. Before further modification, nanorods were centrifuged two times (8000 RPM over 15 min) to remove excess of reactants.

### 2.4. Gold Nanostars (AuNS) Synthesis

Synthesis of gold nanostars was carried out according to the surfactant-free method published by Vo-Dinh et al. [27]. For seed preparation, 15 mL of 1% citrate solution was added to 100 mL of boiling 1 mM HAuCl_4_ solution under vigorous stirring. After 15 min, the mixture was cooled down and kept in the fridge (4 °C) overnight [28]. Then, 100 µL of seeds solution was added to a flask containing 10 mL of 0.25 mM HAuCl_4_ and 10 µL of 1 M HCl, at room temperature, under mild stirring. Quickly after, 30 µL of 10 mM AgNO_3_ and 50 µL of 100 mM ascorbic acid solutions were added simultaneously. The mixture was stirred for 30 s and 1 mL of 1 mM PEG 6k solution was added drop-by-drop. Before further modification, nanostars were centrifuged two times (7000 RPM over 15 min) to remove excess PEG.

### 2.5. Gold Nanotriangles (AuNT) Synthesis

Synthesis of gold nanotriangles was carried out according to a seed–mediated growth method published by Leonardo Scarabelli et al. [29]. For seed preparation, 25 μL of a 50 mM HAuCl_4_ solution was added to 4.7 mL of 100 mM CTAC solution. Subsequently, 300 μL of a freshly prepared 0.01 M NaBH_4_ solution was injected under vigorous stirring. The mixture was kept under mild stirring for 2 h at room temperature to remove excess of borohydride. 

For gold nanotriangles synthesis, two mixtures in vials were prepared: (1) 1.6 mL of 50 mM HAuCl_4_ solution was added to 6.4 mL of 100 mM CTAC solution in 32 mL of Milli-Q water; and (2) 2.35 mL of 10 mM NaI solution was added to solution containing 3.91 mL of 50 mM HAuCl_4_ and 313 mL of 50 mM CTAC. Before proceeding, the initial seed@CTAC solution was diluted 10 times in a 0.1 M CTAC solution. Subsequently, 1.6 mL and 3.13 mL of 100 mM ascorbic acid solution were added to vials 1 and 2, respectively, and both solutions were manually stirred until the complete transparency of the solutions. Finally, 4 mL of diluted seed@CTAC solution was added to vial 1 and stirred manually for 1 s, immediately after solution from vial 1 was added to vial 2 (and manually stirred for few seconds). The mixture was left undisturbed at 30 °C for 1 h. Before further modification, nanotriangles were purified using depletion force method: the CTAC solution was added to reach 200 mM concentration, and the obtained solution was left undisturbed overnight. Then, the supernatant was removed and obtained nanotriangles were re-suspended in water. 

### 2.6. Gold Bipyramids (AuNB) Synthesis

Synthesis of gold nanorods was carried out according to the seed-mediated growth method published by Ana Sánchez-Iglesias et al. [30]. For the preparation of the seeds, 10 mL of 0.25 mM HAuCl_4_ solution was reduced by addition of a freshly prepared NaBH_4_ (0.25 mL, 25 mM) in 50 mM CTAC solution, in the presence of citric acid (5 mM) under vigorous stirring at room temperature. After few minutes of stirring, the vial was closed and the seed solution was heated at 90 °C under mild stirring for 90 min. To prepare the bipyramids, 100 µL of seed solution was added under vigorous stirring to growth solution containing 10 mL of 100 mM hexadecyltrimethylammonium bromide (CTAB), 500 µL of 10 mM HAuCl_4_, 100 µL of 10 mM AgNO_3_, 200 µL of 1 M HCl and 80 µL of 100 mM ascorbic acid. The reaction mixture was left undisturbed for 2 h. Before further modification, bipyramids were centrifuged two times (8000 RPM over 15 min) to remove excess of reactants.

### 2.7. Ligand Exchange Process

Dispersions of isotropic nanoparticles (10 mL of 0.5 mM; rods, triangles, or bipyramids) were centrifuged. Then nanoparticles were re-dispersed in 10 mL of 1 mM PEG-SH 6k solution and left under mild stirring overnight. Afterwards, unbound PEG-SH molecules were removed through centrifugation, supernatant was discarded and precipitate-containing nanoparticles were dispersed in milli-Q water.

To functionalize spherical nanoparticles obtained by Wang’s method, 10 mg of PEG-SH 2k was dissolved in 2 mL CH_2_Cl_2_ and, then 3 mL of 1 mg/mL spherical nanoparticles dispersion was added. The reaction was left under vigorous stirring for over 3 h. Then, the mixture was left undisturbed for 30 min and supernatant was discarded. The precipitate was dispersed in milli-Q water.

### 2.8. Graphene Oxide (GO) Synthesis

Synthesis of graphene oxide was carried out according to a modified Hummers method [31]. Powdered graphite (3 g; with mesh size ca. 20 µm) was added to a solution containing 300 mL 98% H_2_SO_4_ and 30 mL 85% H_3_PO_4_ in an ice bath. Then, 15 g of KMnO_4_ powder was added slowly under mild stirring. After 15 min the solution was brought to 50 °C and exposed to ultrasound for 2 h under vigorous stirring. Then, solution was left overnight under mild stirring. To extract obtained graphene oxide, the solution was slowly poured to a beaker containing 300 mL of distilled water in the form of ice cubes and hydrogen peroxide (10%) was added until the excess potassium permanganate was neutralized. The mixture was left undisturbed for 12 h to allow GO to precipitate and supernatant was discarded. Acidic GO was washed one time with 1% HCl and 4 times with water.

### 2.9. Amide-Functionalized Reduced Graphene Oxide (ARGO) Synthesis

Synthesis of amide–functionalized reduced graphene oxide was carried out according to literature methods [24]. To modify the surface of graphene with amide groups, 250 mg of GO was dried over KOH and dispersed in 200 mL of bis(2-methoxyethyl) ether in an ultrasonic bath. The mixture was quickly heated to 140 °C, and 1.6 mL of N,N-dimethylacetamide dimethyl acetal was added under vigorous stirring. The reaction mixture temperature was increased to 150 °C and left for 24 h, after which it was centrifuged at 3000 RPM. The precipitate was washed once with chloroform, three times with acetone and two times with water.

### 2.10. Carboxyl-Functionalized Reduced Graphene Oxide (CRGO) Synthesis

In order to obtain a carboxylic derivative, amide derivative (ARGO) was dispersed in 400 mL of a 50% water–ethanol solution, and 120 g of KOH was slowly added under vigorous stirring. The reaction mixture was kept in reflux for 48 h. The product was washed with water until the pH became neutral, and was dispersed in 100 mL of water.

### 2.11. Pristine Reduced Graphene Oxide (RGO) Synthesis

Reduced graphene oxide without carboxyl group functionalization was obtained in a similar process to that of the amide derivative (ARGO) except the addition of N,N-dimethylacetamide dimethyl acetal. Briefly, 250 mg of graphene oxide dried over KOH was dispersed in 200 mL of bis(2-methoxyethyl) ether in an ultrasonic bath and the solution was quickly brought to 150 °C and left for 24 h. Then, the reaction mixture was cooled down and centrifuged at 3000 RPM, precipitate was washed with chloroform, three times in acetone and two times in water.

### 2.12. G/NP Composite Synthesis

In a typical process, 500 µL ca. 0.1 mg/mL of graphene dispersion was added to (0.5 mM Au(0)) dispersion of PEG-modified nanoparticles and left under mild stirring overnight. To tune the density of nanoparticles grafting, we varied amount of the added nanoparticles dispersion. In the case of spherical nanoparticles, the amounts were 5, 80, and 400 µL for CRGO@AuNP_1, CRGO@AuNP_2, and CRGO@AuNP_3, respectively. For the anisotropic nanoparticles, the gold-to-graphene ratio was increased to compensate for their much higher volume vs. area ratio; the amounts were 533 µL (for low coverage, e.g., sample CRGO@AuNR_1, see Section 3 for details) and 2,665 µL (for high coverage, e.g., sample CRGO@AuNR_2, see Section 3 for details).

### 2.13. Nanoparticle Density Analysis

To evaluate the number of nanoparticles per graphene surface area, transmission electron microscopy TEM images of the CRGO sample with nanoparticles were analyzed using ImageJ software built-in function “Analyze Particles” that returns the area (in our setup in nm^2^) for each detected particle/aggregate of particles by counting all connected pixels with specified color intensity. To remove entries produced by noise and smaller objects that were detected by ImageJ (version 1.52k 29 January 2019, Wisconsin, USA), the average surface area (in nm^2^) of a single nanoparticle was calculated—relatively clean (no objects except the nanoparticles) part of the original image containing 30 to 50 nanoparticles was subject to the same procedure in ImageJ as the full image and total surface area of said nanoparticles was used to calculate the average surface area of a single nanoparticle. Then, data from full image was filtered by removing all entries with surface area smaller than average surface area of a single nanoparticle times a factor of 0.5, which left us only with entries produced by single nanoparticles and aggregates of two or more nanoparticles (Figure S1). Using total surface area of nanoparticles, average surface area of single nanoparticle and surface of graphene on the full image (calculated using ImageJ built-in function “Measure polygon area”), we calculated the number of nanoparticles per graphene area for each G/NPs sample.

### 2.14. Structural Analyses of Composite Materials

The obtained materials were subjected to structural analysis using transmission electron microscopy (TEM, Warsaw, Poland) Zeiss Libra 120 microscope, with LaB6 cathode, equipped with OMEGA internal columnar filters (Carl Zeiss NTS GmbH, Germany) and CCD camera (Carl Zeiss NTS GmbH, Germany), available at Faculty of Chemistry of the University of Warsaw, TEM model JEM–1400 (JEOL, Japan), available in Nencki Institute of Experimental Biology, laboratory of electron microscopy, TEM model JEM–1011 (JEOL) equipped with a model EDS INCA analyzer (Oxford, UK), in the Electron Microscopy Platform, Massakowski Medical Research Centre Polish Academy of Science Warsaw and scanning electron microscope (SEM, Warsaw, Poland) Zeiss LEO 435VP with tungsten cathode available at the Faculty of Chemistry University of Warsaw (Figure S2–S5). Preparation of samples included a drop-casting 5 µL of graphene/nanoparticles water solution on TEM grid (for transmission electron microscope) or on silicon wafer (for scanning electron microscope) and subsequent drying on air and then under vacuum. For further structural analysis of the obtained materials, the atomic force microscope technique (AFM, Warsaw, Poland) was introduced. AFM Bruker (Billerica, USA), Dimension Icon available at the Biological and Chemical Research Centre, University of Warsaw was used. In order to analyze the elemental composition, X-ray photoelectron spectroscopy method (XPS, Warsaw, Poland) was employed. Characterization was performed using a PHI 5000 VersaProbe (ULVAC-PHI, Chanhassen, USA) located at Laboratory of Surface Analysis–Institute of Physical Chemistry Polish Academy of Sciences. Sample preparation for XPS was identical to previously the described protocol for SEM sample preparation. Spectroscopy in the UV-visible range (UV-VIS) studies were performed using UV-3600 Plus UV-Vis-NIR spectrophotometer (Duisburg, Germany) available at the University of Warsaw. FT-IR spectra were collected for KBr pellets doped with small amount of CRGO material using Shimadzu FTIR-8400S equipment (Duisburg, Germany).

## 3. Results and Discussion

### 3.1. Carboxyl-Modified Reduced Graphene Oxide

In this study, we relied on two important facts to develop an ex situ method for reduced graphene oxide decoration: (1) the advantages of non-covalent methods of G/NP composite material formation, which ensures high flexibility and versatility in designing these hybrid materials; and (2) that the most popular protocols for nanoparticle synthesis are performed in water, especially for anisotropic nanoparticles [32]. Considering both, we excluded pristine reduced graphene oxide as the potential 2D platform, since such rGO does not bear functional groups (or in the best case a very limited number of functional groups), making it insoluble in water. Thus, we decided to prepare a functionalized derivative of rGO. We chose a perspective structure, the carboxyl-functionalized reduced graphene oxide, previously shown to form stable suspensions in aqueous solutions [24], even distribution of functional groups throughout the 2D surface, as well as being a good template for creating hybrid materials [33]. Moreover, we assumed that carboxyl moieties are good candidates for introducing non-covalent interactions with nanoparticles. Along this line, we prepared graphene oxide using a modified Hummers method [31], and then performed a Eschenmoser–Claissen sigmatropic rearrangement reaction onto its surface to install amide functionalities. Finally, we converted amides to carboxyl salts through hydrolysis.

**Figure 1:**
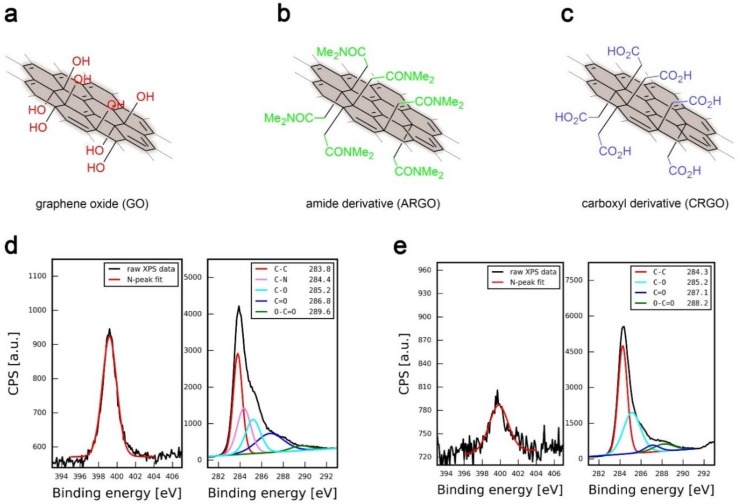
Schematic representation of graphene materials: (**a**) graphene oxide (GO), (**b**) amide derivative of reduced graphene oxide (ARGO), and (**c**) final product, carboxyl derivative of reduced graphene oxide (CRGO). Along the structures, results of high resolution X-ray photoelectron spectroscopy (XPS) analysis of nitrogen and carbon peak regions are shown for (**d**) amide derivative (ARGO) and (**e**) carboxyl derivative (CRGO) of reduced graphene oxide.

To confirm successful decoration of reduced graphene oxide with carboxyl moieties and evaluate their amount on the surface of CRGO material, XPS analysis was performed. Beyond the final product, analyses of graphene oxide and amide derivatives of graphene oxide (ARGO) was also performed to follow the evolution of these materials throughout synthesis. The C(1s) peak for ARGO (Figure 1d) material was deconvoluted, and five distinctive peaks were found at 283.8 eV, 284.4 eV, 285.2 eV, 286.8 eV, and 289.6 eV corresponding to chemically different carbon atoms: C-C, C-N, C-O, C=O, and O-C=O, respectively [34,35]. Similar chemistries of carbon atom were found after deconvolution of the CRGO C(1s) peak (Figure 1e).

High resolution XPS analysis of ARGO revealed a peak around 400 eV, indicating nitrogen presence in the material at 4.25 atomic percent. This result evidences that the Eschenmoser–Claisen sigmatropic rearrangement reaction was successful and amide functionalities were installed on the surface of a 2D material. However, the same peaks, but with much lower intensities, were found in both GO and CRGO samples, indicating nitrogen presence at 0.55 atomic percent for each sample (see Figure S6 for more details). Since both GO and CRGO samples, in principle, should not contain any nitrogen atoms in their structure, it was assumed that the peaks presence was due to nitrogen contamination, most likely from atmospheric pick-up [36]. To increase the precision of calculation for the amide groups introduced in the ARGO material, it was assumed, that the same amount of atmospheric nitrogen was physisorbed on the ARGO sample.

Given the above, the N-to-C ratio in the amide-functionalized RGO was estimated to be 0.052, which can be translated to one amide moiety (-CH_2_-CON(CH_3_)_2_) per ca. 18 carbon atoms in graphene structure. In other words, 1 amide group is present per ca. 1 nm^2^ of a single layer graphene material. Consequently, the same density of carboxyl groups should be found for CRGO, assuming full carboxyl-to-amide conversion during hydrolysis. These values are in good agreement with previous investigations [24].

**Figure 2:**
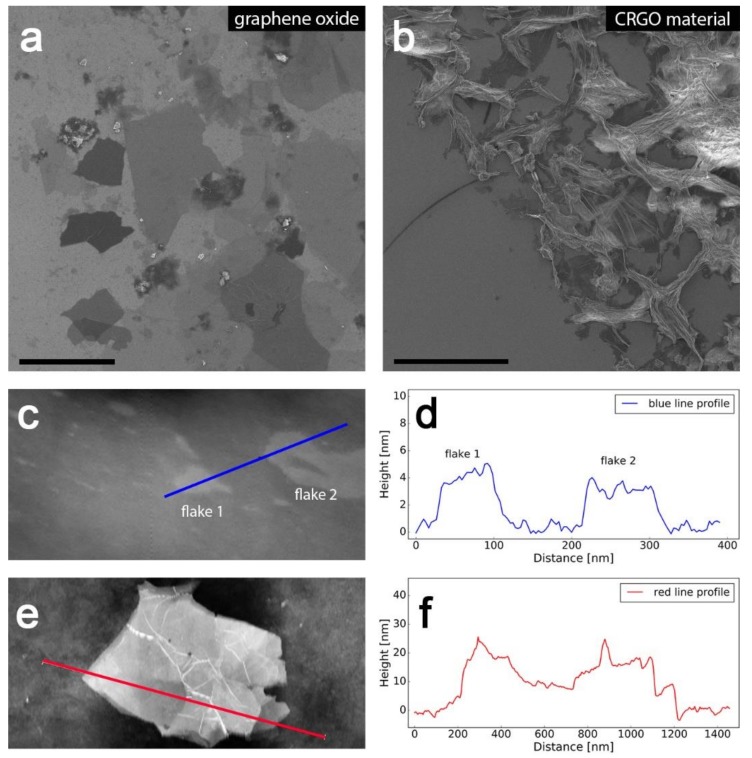
Microscale investigation of graphene materials. (**a**) Scanning electron microscope (SEM) images of graphene oxide (**b**) final carboxyl derivative along with AFM images, and (**c**) the corresponding height profiles of CRGO (**c**–**f**). Scale bars in panels (**a**,**b**) correspond to 10 µm.

To get a more detailed picture of the structure of graphene materials, scanning electron microscope analysis was performed. Figure 2a–b shows SEM images of GO and final CRGO material. To obtain insight into the 3D structure of CRGO material, atomic force microscopy analysis was performed, which showed that the material consisted mainly of few-layered graphene (Figure 2c–d) along with fraction of single-layered graphene (Figure 2e–f), as can be expected when obtaining reduced graphene oxide through the Hummers method [37].

**Figure 3:**
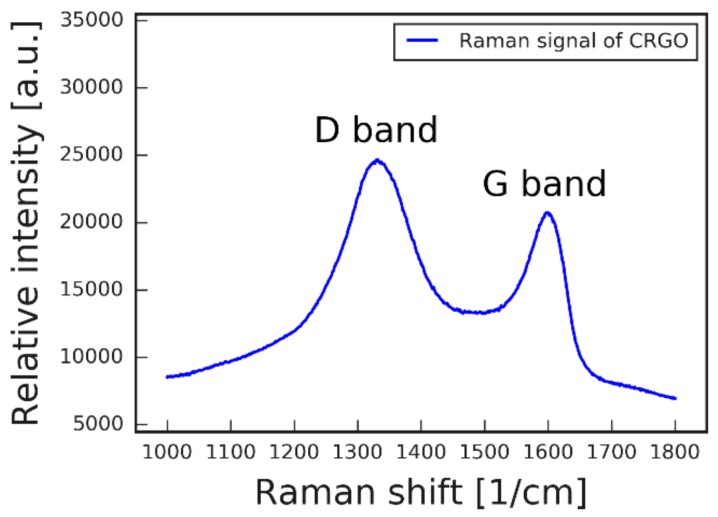
Raman spectrum of CRGO showing the presence of strong D- and G-bands.

To further confirm the structure of the final graphene product, Raman and FTIR analyses of CRGO were performed. Figure 3 shows a chosen region of the collected Raman spectrum that reveals signals characteristic to in-plane sp^2^ C–C bond stretching (G-band) and defects in sp^2^ carbon systems (D-band) [38]. As expected, a strong D-band signal was present due to high amount of defects in graphene sheet. Namely, a relatively large number of sp^3^ atoms within the graphene sheet were present due to functionalization with carboxylic moieties. This result is in line with XPS analysis results, which evidenced that carboxylic groups were installed throughout the graphene sheet. The FTIR data (Figure S7) provide additional insight into the functional groups of the material. Characteristic peaks for O–H stretching (broad 3,500 1/cm) and bending (1,641 and 1,375 1/cm) were identified, coming from carboxyl groups, small portion of residual hydroxyl groups, and water adsorbed on graphene. Importantly, we noted the presence of a 1,709 1/cm peak, which can be attributed to C=O stretching of carboxyl moieties, confirming their presence in CRGO material [39]. The acquired spectra were in good agreement with previous analysis of analogous material [24], and the XPS analysis discussed above. 

### 3.2. Metal Nanoparticles

As the inorganic part of G/NPs hybrid materials we decided to use a variety of nanoparticles with different shapes and sizes. The ability of incorporating many different types of nanoparticles allows one to precisely determine properties of the final hybrid material, since properties (and perspective applications [40]) of nanoparticles strongly depend on their architecture. 

**Figure 4:**
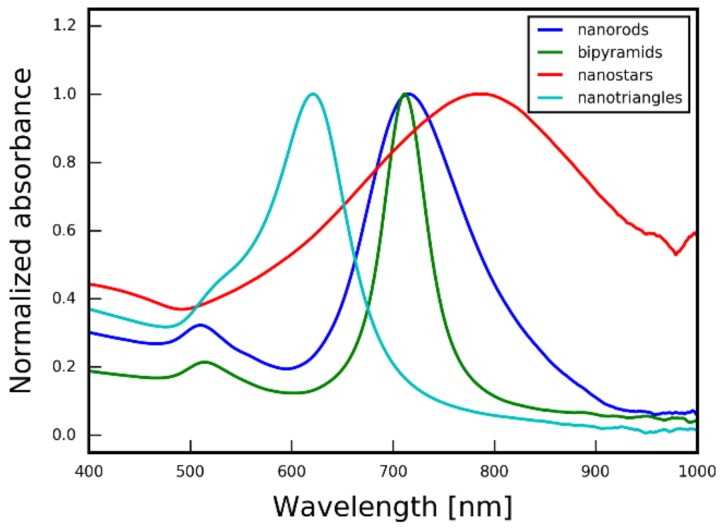
Normalized UV-VIS absorption spectra of gold nanorods, bipyramids, stars, and triangles dispersion. Transmission electron microscope images of these nanoparticles are given in Figure 5.

Gold nanorods (AuNR), nanospheres (AuNP), nanostars (AuNS), nanotriangles (AuNT), and bipyramids (AuNB) were prepared using literature methods (for details please see the Section 2). The size and monodispersity of gold spherical nanoparticles were determined using TEM analysis, confirming successful synthesis of 5.1 ± 0.85 nm particles. Obtained anisotropic nanoparticles were characterized by UV-VIS spectrophotometry. Figure 4 shows the UV-VIS spectra of nanorods, bipyramids, nanostars, and nanotriangles with different localized surface plasmon resonance (LSPR) peak positions. Narrow peaks for rods, triangles, and bipyramids confirmed their monodispersity and relative lack of impurities [41].

**Figure 5:**
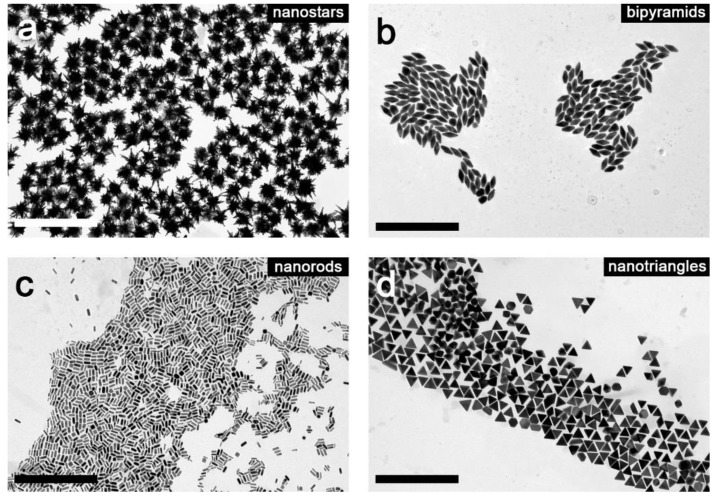
TEM micrographs of anisotropic gold nanoparticles: nanostars (**a**), bipyramids (**b**), nanorods (**c**), and nanotriangles (**d**). The scale bars on each micrograph represent 500 nm.

Transmission electron microscope analysis of all anisotropic nanoparticles was also performed (Figure 5), confirming synthetic success and providing insight into their sizes and monodispersity which were evaluated accordingly: bipyramids, 93.3 and 38.9 nm (length and width, respectively); nanorods, 59.2 and 20.7 nm (length and width, respectively); nanotriangles, 70.5 nm (edge length); and nanostars, 103.7 nm (diameter, including spikes). The size distribution of measurements was less than 10% for all types of nanostructures.

To ensure compatibility with the carboxyl-modified graphene surface, PEG-SH ligand was chosen as a capping agent for all types of nanoparticles. The thiol group affinity to the gold surface [42] ensures high stability of functionalized nanoparticles, even without the presence of uncapped, free ligand in the solution. This provides the means for removing excess ligands without causing the aggregation of nanoparticles (see Section 2 for experimental details). The PEG chains are responsible for attaching the nanoparticles to carboxyl-modified surface of graphene through formation of hydrogen bonds and van der Waals interactions. Ligand exchange reactions were performed using standard literature protocols [43]. Afterwards, nanoparticles were purified to remove excess of free ligands.

### 3.3. G/NPs Composites Based on Carboxyl-Functionalized Reduced Graphene Oxide and Spherical Gold Nanoparticles

**Figure 6:**
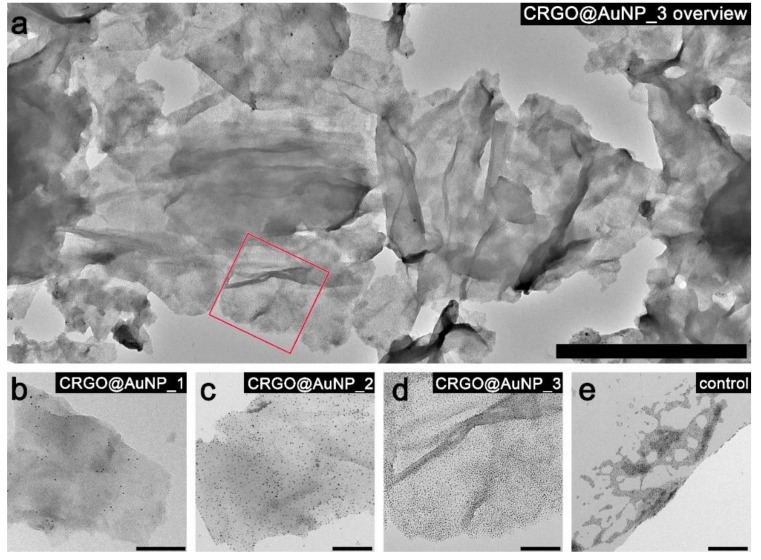
TEM micrographs of different graphene–spherical nanoparticles composites. (**a**) A zoom-off image of GNP-2 material showing several graphene sheets decorated with nanoparticles. (**b**–**d**) Zoom-in TEM images taken for GNP-1, GNP-2, and GNP-3 materials, respectively. Scale bar in image (**a**) represents 2 µm and in images (**b**–**e**) 200 nm. The red square in image (**a**) corresponds to the area of the sample shown in panel (**d**).

The above described carboxyl-functionalized graphene and spherical nanoparticles were subject to preparing hybrid materials. For this purpose, we prepared mixtures of these materials and allowed nanomaterials to interact for 12 h under gentle stirring (see Section 2 for details). The CRGO/AuNP ratio was varied to test if it is possible to control density of nanoparticle grafting. Figure 6a–d shows TEM images of CRGO material covered with PEG-modified spherical nanoparticles. Notably, nanoparticles were randomly distributed over the entire surface of graphene, while they were not present outside of the graphene area, confirming successful preparation of composites, and allowing to conclude that interactions between nanoparticles and modified graphene surface are sufficiently strong to decorate graphene. In a control experiment with pristine reduced graphene oxide (RGO) used in the place of CRGO, we noticed that nanoparticles tended to form aggregates instead of adsorbing evenly on graphene (Figure 6e). This effect can be attributed to the absence of carboxyl groups, which prevents efficient interactions between the nanoparticles, PEG ligand and graphene surface, confirming proper choice of the graphene platform.

A series of images shown in Figure 6b–d exemplify that with the increasing number of nanoparticles used in the preparation process, increasing coverage density of NPs on graphene can be observed. To quantify these observations, a thorough, semi-automated analysis of the images was performed (details are given in Section 2). The number of nanoparticles was estimated to be around 200 NPs per µm^2^ for CRGO@AuNP_1, 3000 NPs/µm^2^ for sample CRGO@AuNP_2 and 14,000 NPs/µm^2^ for CRGO@AuNP_3.

### 3.4. G/NPs Composites Based on Carboxyl-Functionalized Reduced Graphene Oxide and Anisotropic Gold Nanoparticles

**Figure 7:**
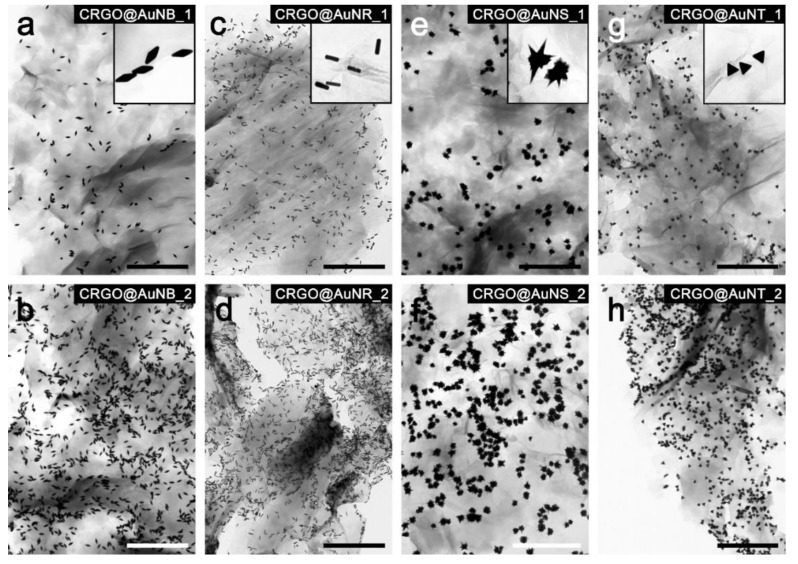
TEM micrographs of GNP composites based on anisotropic nanoparticles. Samples obtained using: (**a**,**b**) bipyramid; (**c**,**d**) nanorods; (**e**,**f**) nanostars; and (**g**,**h**) nanotriangles. Two densities of grafting are shown: lower and higher in upper and lower rows, respectively. Insets in panels (**a**,**c**,**e**,**g**) show magnified areas of the composites to clearly evidence shapes of nanoparticles. Additional images of these composites are shown in Figures S2-S5. Scale bars correspond to 1000 nm.

The above-described methodology of G/NP composites’ hybrid formation was then applied to anisotropic nanoparticles. Figure 7 shows TEM micrographs of graphene covered with these nanoparticles. Four different shapes of nanoparticles (triangles, stars, rods and bipyramids) are presented. For each NP type we aimed at achieving two densities of coverage. 

**Figure 8:**
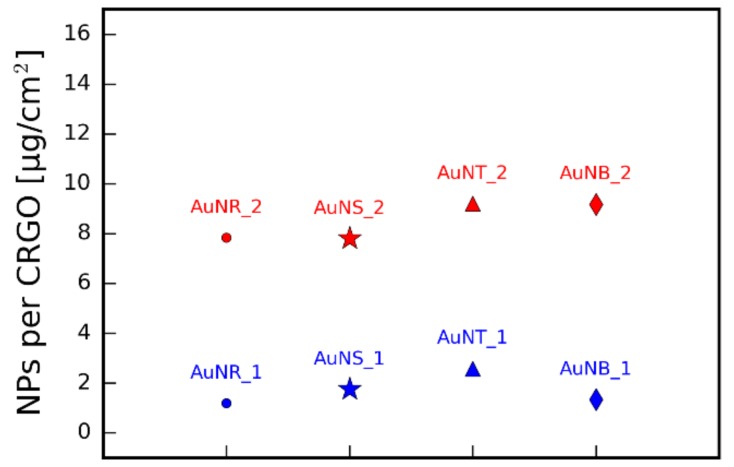
The estimated amount of gold per graphene area for G/NPs composites based on anisotropic nanoparticles.

Prior to the deposition process, anisotropic nanoparticles were functionalized with PEG-SH ligand and the hybrid material formation occurred in the same manner as previous samples with spherical nanoparticles. Notably, differing the size and shape of nanoparticles did not alter their ability to absorb onto the CRGO surface. This confirms that the driving force behind composites formation is dependent on chemical properties (and thus affinity to carboxyl groups on the graphene surface) of the nanoparticles, which are defined by surface ligands. In a similar fashion to spherical nanoparticles, the presented method allows the deposition of anisotropic nanoparticles at different densities. Quantitative analysis is presented in Table 1 and Figure 8.

The density of coverage, after conversion to nanoparticles mass (in mg of Au) per graphene area, remained roughly at the same levels for each sample with lower (Figure 8, blue markers) and higher (Figure 8, red markers) nanoparticle densities. This result is consistent with the preparation procedure. In each case of lower and higher number of nanoparticles, the gold-to-graphene ratio was kept the same (ca. 1 mg Au per 1 mg CRGO for lower densities and ca. 5 mg Au per 1 mg CRGO for higher densities). These results further confirm that the deposition process is independent on the metallic core of NPs and has similar efficiency.

### 3.5. Stability Test of CRGO@AuNP

**Figure 9:**
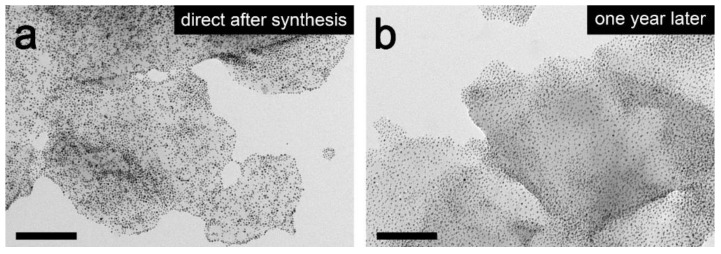
Micrographs of the same sample of CRGO@AuNP hybrid material directly after synthesis (**a**) and a year after (**b**). Scale bars represents 200 nm.

To verify the stability of the obtained materials, TEM analysis of the same CRGO@AuNP composite sample was performed directly after obtaining the material (Figure 9a) and one year after (Figure 9b), no signs of degradation of the material, e.g., coalescence of nanoparticles or change in nanoparticles coverage on graphene (which would suggest lowering of CRGO/NPs interactions) were evidenced, confirming the material’s long-term stability, even when stored under ambient conditions (25 °C, air atmosphere). 

## 4. Conclusions

In summary, we show that rational design of surface modification of nanomaterials is an efficient way to prepare composites. Namely, we introduced carboxyl groups throughout the entire surface of reduced graphene oxide and polyethylene glycol ligands on metallic nanoparticle surfaces. These modifications allowed us to prepare a set of G/NP composite materials by simply mixing the prepared constituents. Control experiments indicate that these structures form through hydrogen bonding and Van der Waals forces. We have shown that our approach is versatile, namely it can be applied to create graphene composites with nanoparticles with various morphologies (spheres, rods, triangles, stars, and bipyramids), and a wide range of sizes, confirming that PEG ligands efficiently screen the metallic core of nanoparticles. Furthermore, we showed that we can control the density of nanoparticle grafting for each type of nanoparticles and that hybrid materials obtained by our technique are stable over extended periods of time. We anticipate that simplicity, versatility, and stability of the composites will make this method appealing for various researchers aiming to prepare and utilize graphene-nanoparticles composites in the areas that include improved selective electrochemical detection [44] as well as energy conversion (e.g., photocatalysis [45] or fuel cells technology [46]).

## Figures and Tables

**Table 1:** Estimation of anisotropic nanoparticles’ density on graphene.

Sample	Image	NPs [1/µm^2^]	NPs [µg/cm^2^]
CRGO@AuNB_1	Figure 7a	13	1.33
CRGO@AuNB_2	Figure 7b	87	9.17
CRGO@AuNR_1	Figure 7c	25	1.29
CRGO@AuNR_2	Figure 7d	166	7.83
CRGO@AuNS_1	Figure 7e	10	1.75
CRGO@AuNS_2	Figure 7f	43	7.80
CRGO@AuNT_1	Figure 7g	26	2.59
CRGO@AuNT_2	Figure 7h	94	7.20

## Supplementary Materials

The following are available online at https://www.mdpi.com/2079-4991/9/4/602/s1, Figure S1. Example analysis of nanoparticle density on sample CRGO@AuNB_1 (a,c) and CRGO@AuNB_2 (b,d). Red marker on chart a and b indicates where the measured object’s area becomes smaller than average bipyramid surface area times 0.5. Green lines on TEM images c and d indicates graphene area taken into account during density calculation, while red lines mark areas completely excluded due to contrast/quality issues. See Section 2 of the main text for more details.; Figure S2. SEM micrograph of gold nanorods on CRGO.; Figure S3. SEM micrograph of gold bipyramids on CRGO.; Figure S4. SEM micrograph of gold nanostars on CRGO.; Figure S5. SEM micrograph of gold nanotriangles on graphene.; Figure S6. XPS spectra of GO, ARGO, and CRGO along with calculated elemental composition.; Figure S7. FT-IR spectra of CRGO.
