# Supplementary material for: Universal Method for Producing Reduced Graphene Oxide/Gold Nanoparticles Composites with Controlled Density of Grafting and Long-Term Stability
Source: Nanomaterials (Basel). 2019 Apr 11;9(4):602. doi: 10.3390/nano9040602 (PMC6523825; doi:10.3390/nano9040602)
Supplement: Supplementary file 1 [file nanomaterials-09-00602-s001.pdf]

# Universal method for producing reduced graphene oxide/nanoparticles composites with controlled density of grafting and long-term stability.

## SUPPORTING INFORMATION

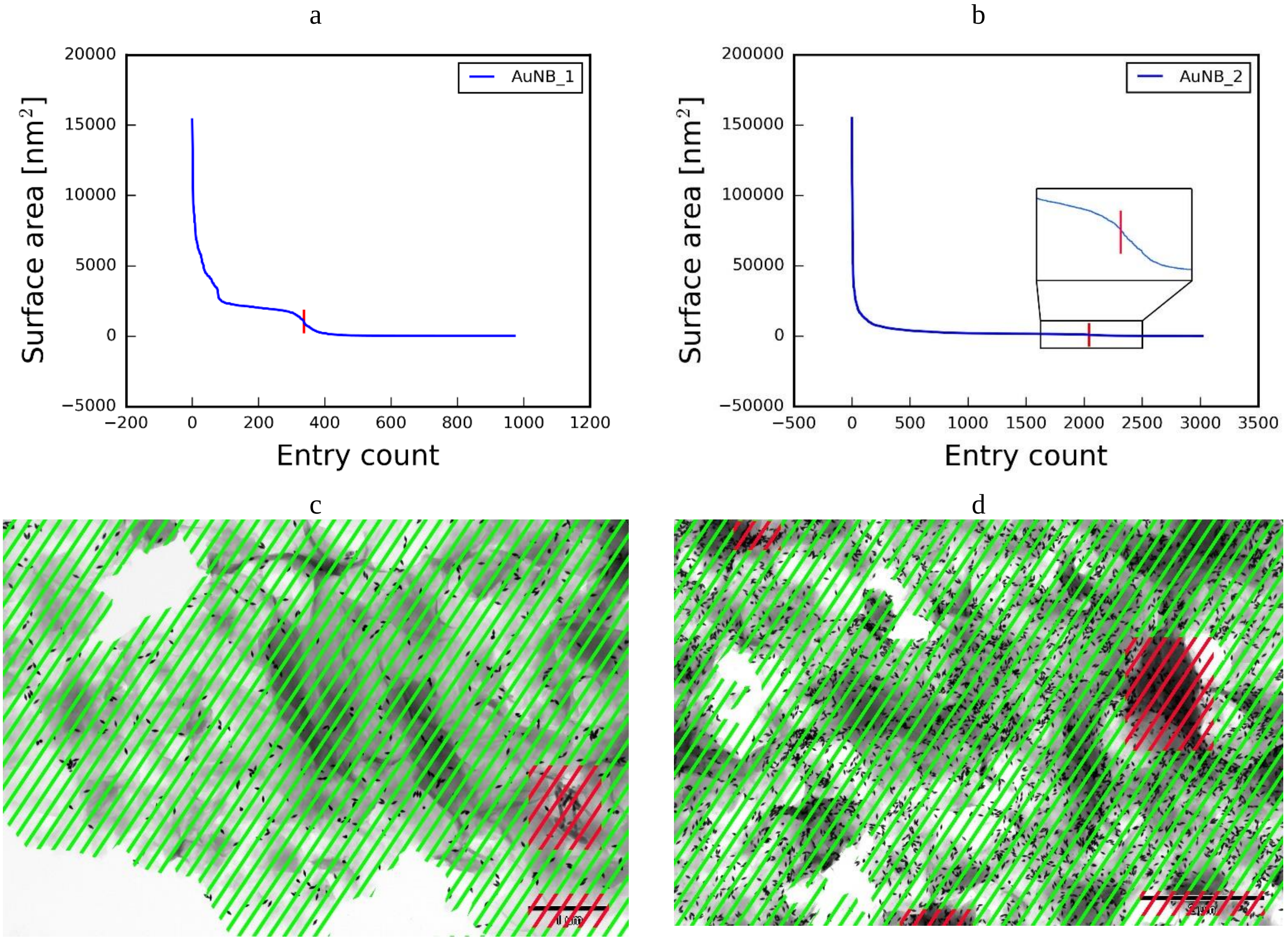


**Figure S1.** Example analysis of nanoparticle density on sample CRGO@AuNB_1 (a, c) and CRGO@AuNB_2 (b, d). Red marker on chart a and b indicates where measured object's area becomes smaller than average bipyramid surface area times 0.5. Green lines on TEM images c and d indicates graphene area taken into account during density calculation while red lines mark areas completely excluded due to contrast/quality issues. See materials and methods section of the main text for more details.

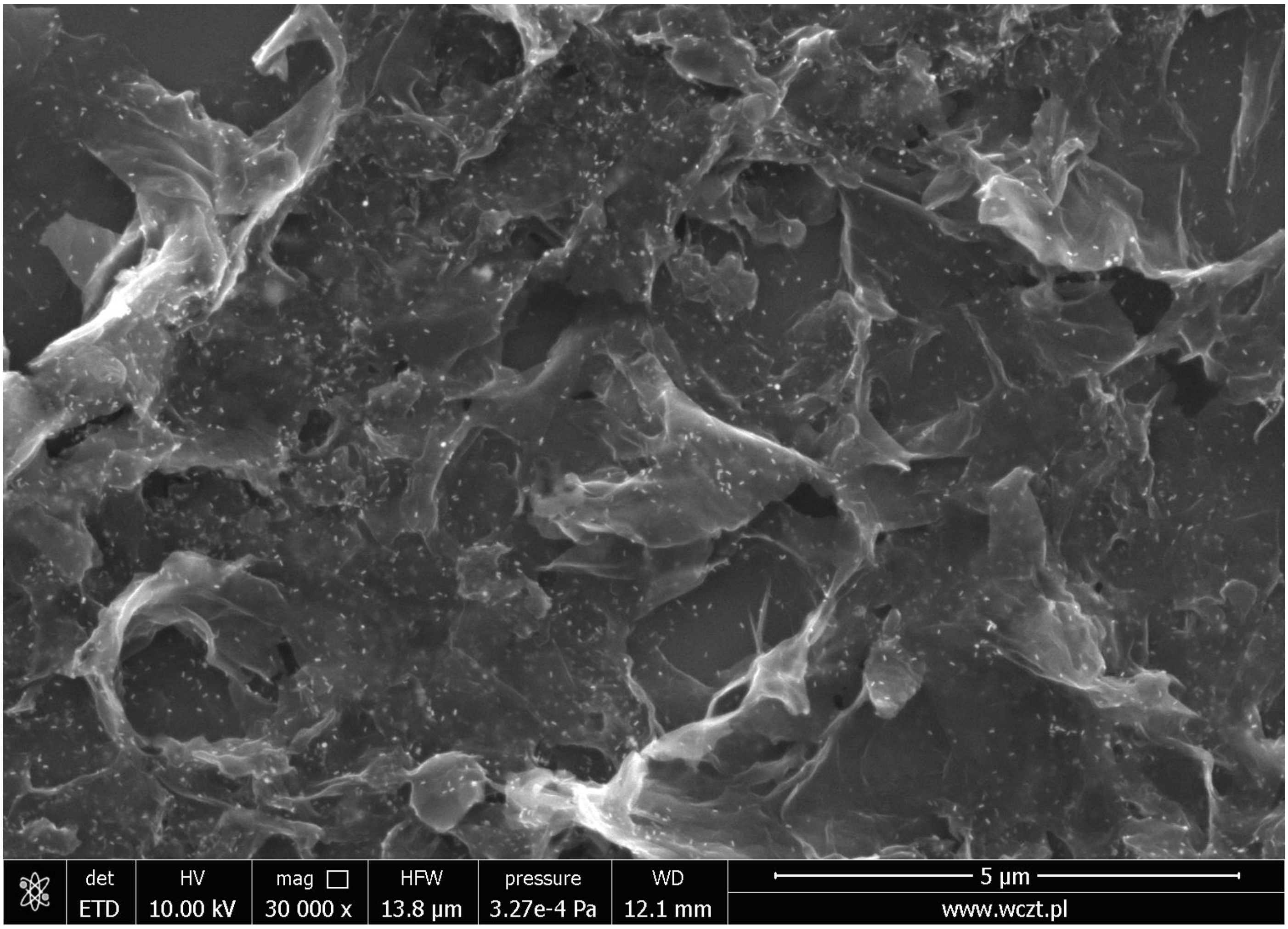


**Figure S2.** SEM micrograph of gold nanorods on CRGO

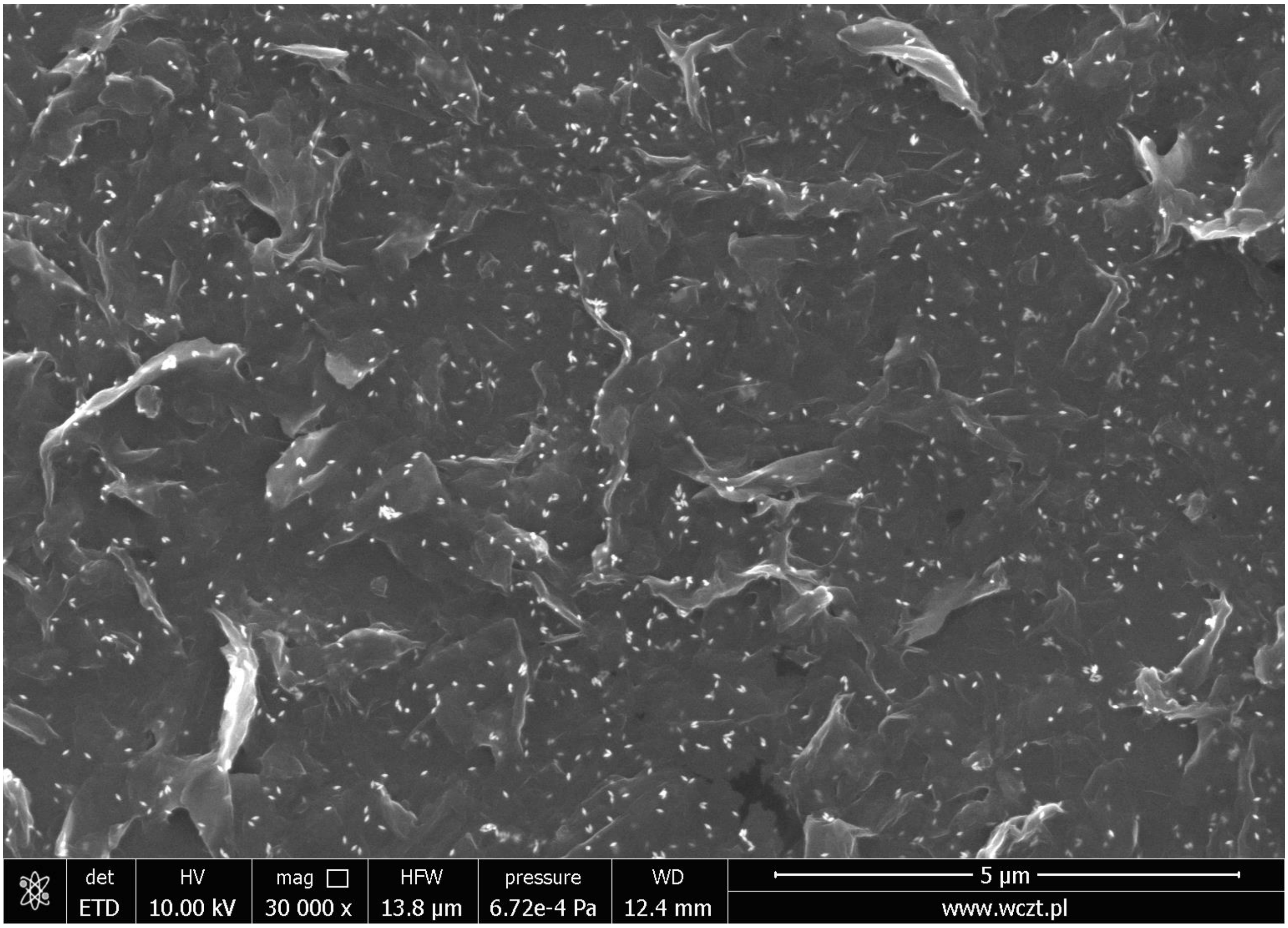


**Figure S3.** SEM micrograph of gold bipyramids on CRGO

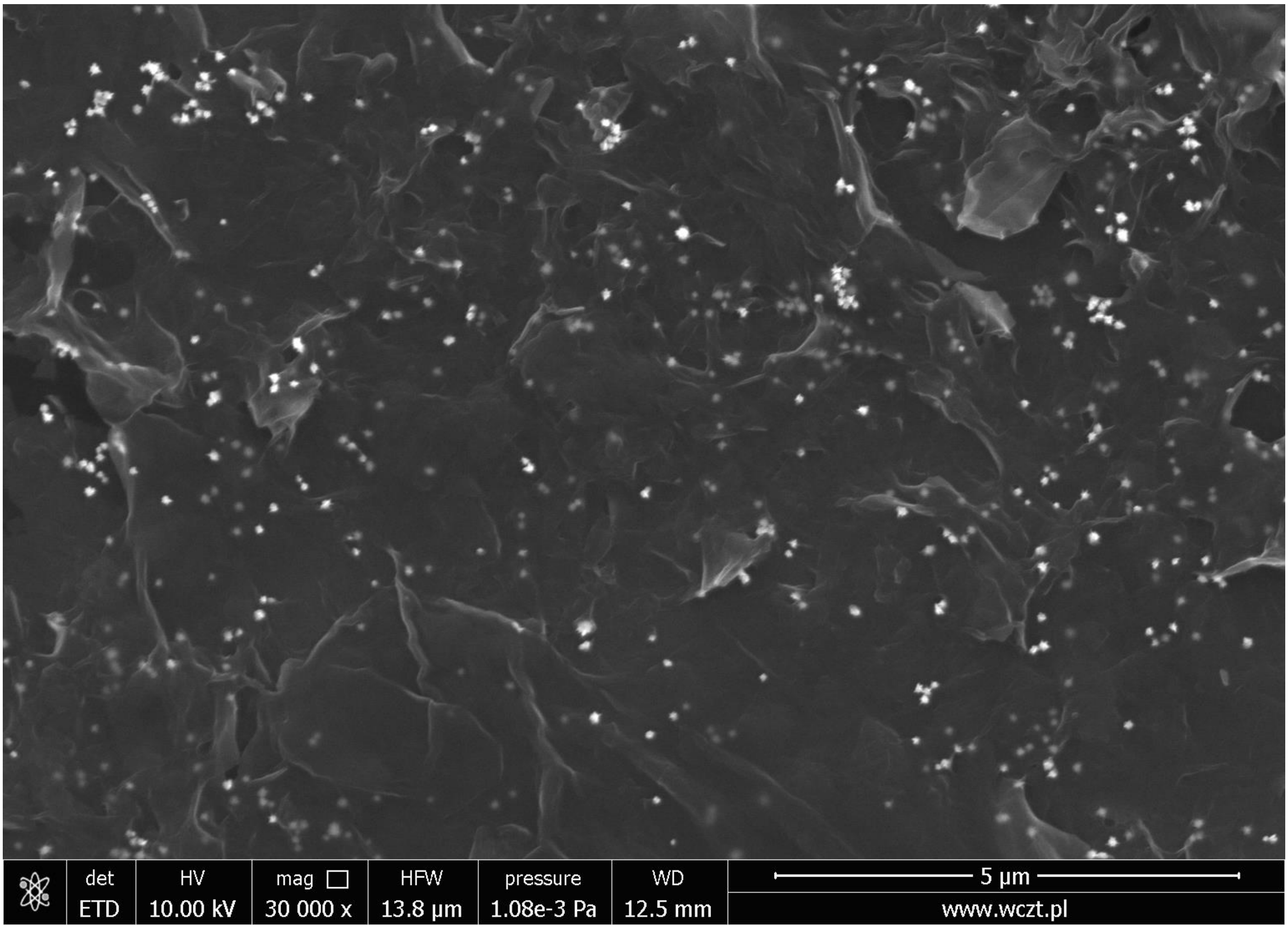


**Figure S4.** SEM micrograph of gold nanostars on CRGO

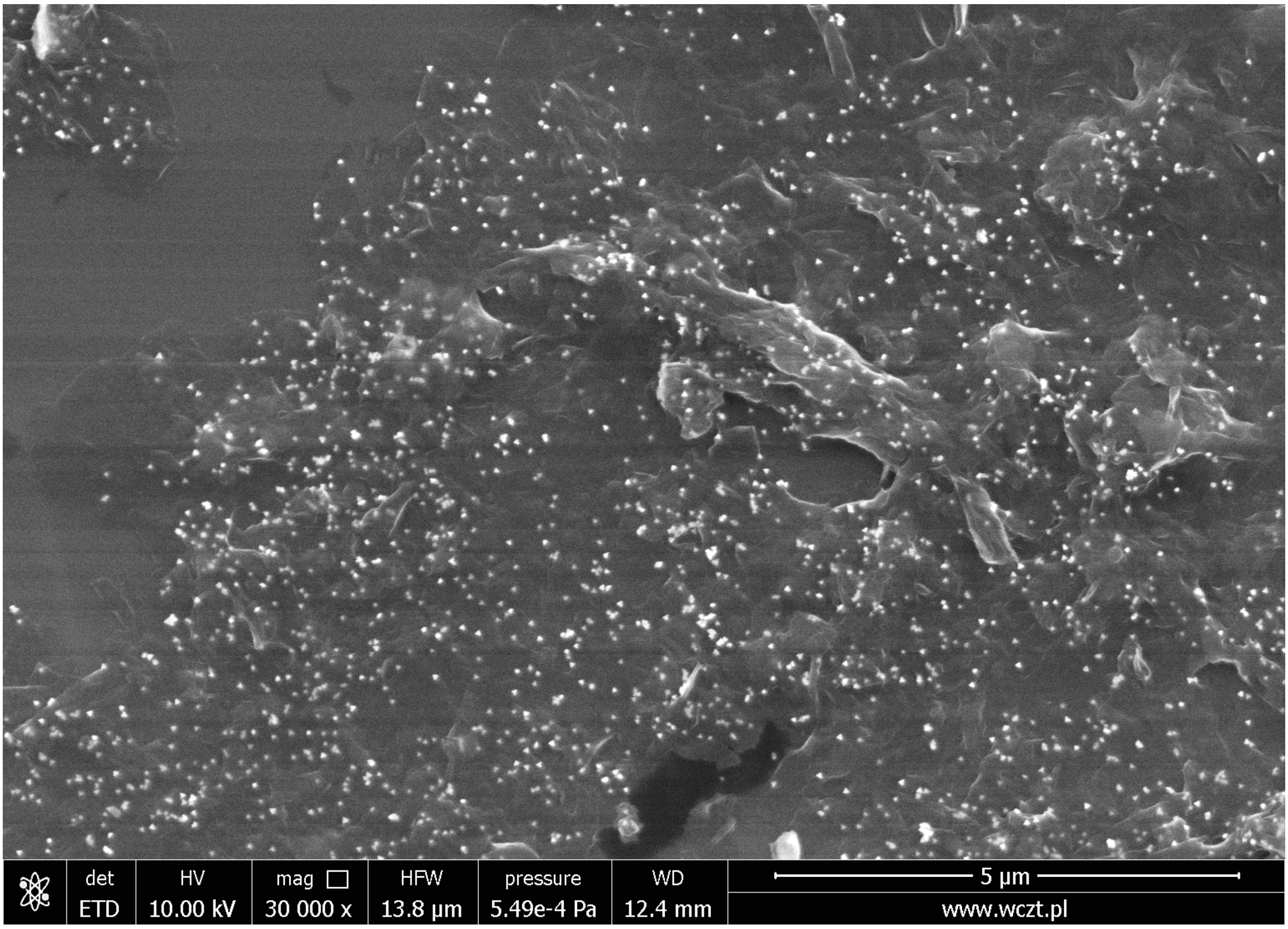


**Figure S5.** SEM micrograph of gold nanotriangles on graphene

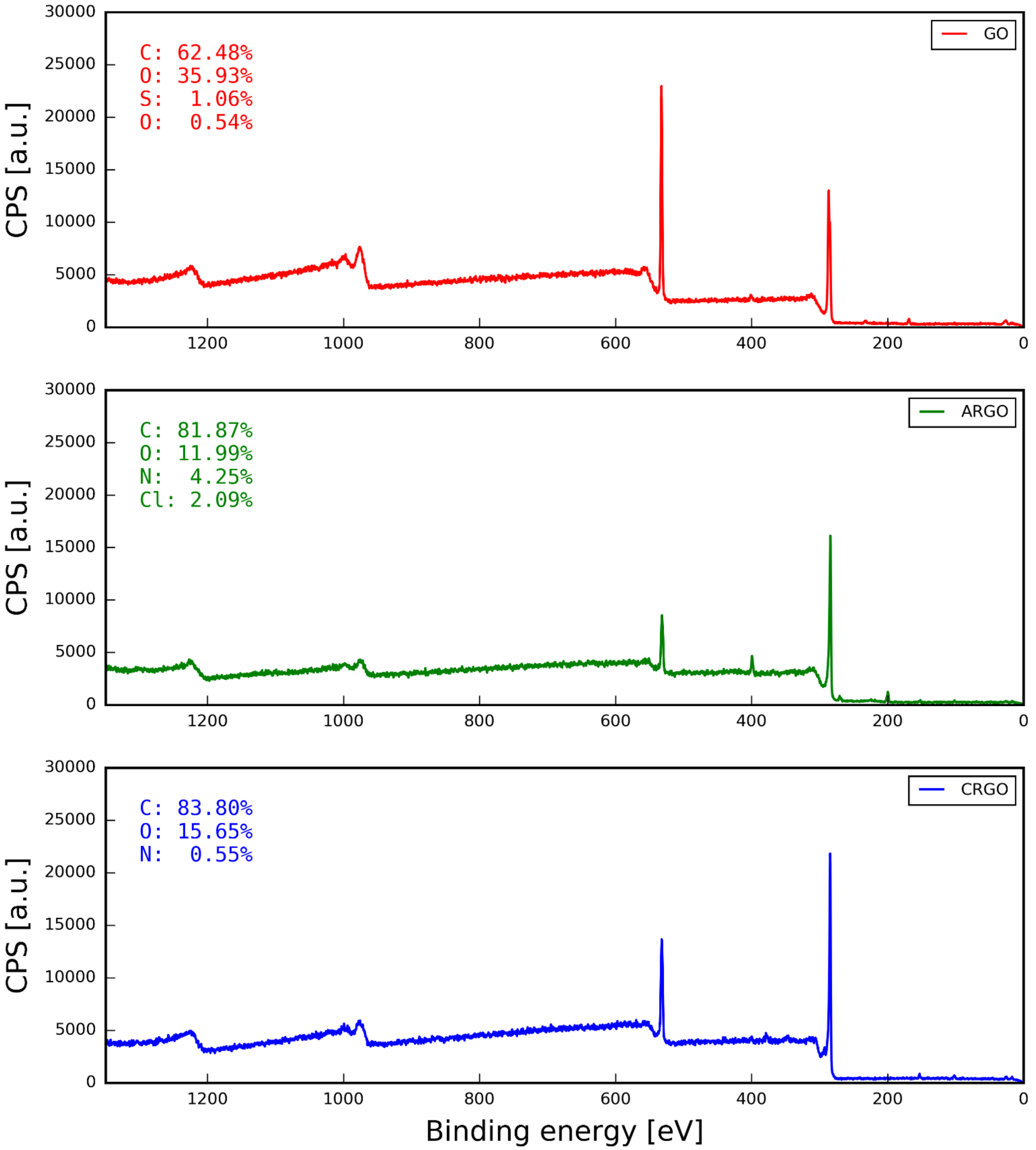


**Figure S6.** XPS spectra of GO, ARGO and CRGO along with calculated elemental composition.

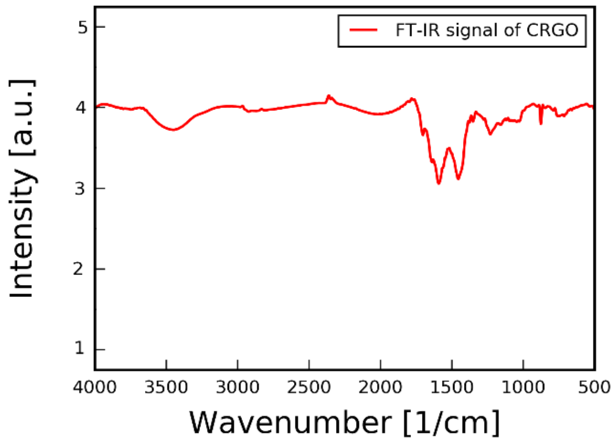


**Figure S7.** FT-IR spectra of CRGO.
